# Measuring group function in problem-based learning: development of a reflection tool

**DOI:** 10.1186/s12909-023-04726-y

**Published:** 2023-10-10

**Authors:** Athena Li, Matthew Mellon, Amy Keuhl, Matthew Sibbald

**Affiliations:** 1 Faculty of Health Sciences, McMaster University, Hamilton, Canada; 2 Michael G. DeGroote School of Medicine, McMaster University, Hamilton, Canada; 3 McMaster Education Research, Innovation & Theory (MERIT) Program, McMaster University, Hamilton, Canada; 4 Department of Medicine, McMaster University, Hamilton, Canada

**Keywords:** Curriculum design, Problem-based learning

## Abstract

**Background:**

Problem-based learning (PBL) is a pedagogy involving self-directed learning in small groups around case problems. Group function is important to PBL outcomes, but there is currently poor scaffolding around key self-reflective practices that necessarily precedes students’ and tutors’ attempts to improve group function. This study aims to create a structured, literature-based and stakeholder-informed tool to help anchor reflective practices on group function. This article reports on the development process and perceived utility of this tool.

**Methods:**

Tool development unfolded in four steps: 1) a literature review was conducted to identify existent evaluation tools for group function in PBL, 2) literature findings informed the development of this new tool, 3) a group of PBL experts were consulted for engagement with and feedback of the tool, 4) four focus groups of stakeholders (medical students and tutors with lived PBL experiences) commented on the tool’s constructs, language, and perceived utility. The tool underwent two rounds of revisions, informed by the feedback from experts and stakeholders.

**Results:**

Nineteen scales relating to group function assessment were identified in the literature, lending 18 constructs that mapped into four dimensions: Learning Climate, Facilitation and Process, Engagement and Interactivity, and Evaluation and Group Improvement. Feedback from experts informed the addition of missing items. Focus group discussions allowed further fine-tuning of the organization and language of the tool. The final tool contains 17 descriptive items under the four dimensions. Users are asked to rate each dimension holistically on a 7-point Likert scale and provide open comments.

Researchers, faculty, and students highlighted three functions the tool could perform: (1) create space, structure, and language for feedback processes, (2) act as a reference, resource, or memory aid, and (3) serve as a written record for longitudinal benchmarking. They commented that the tool may be particularly helpful for inexperienced and poor-functioning groups, and indicated some practical implementation considerations.

**Conclusion:**

A four-dimension tool to assist group function reflection in PBL was produced. Its constructs were well supported by literature and experts. Faculty and student stakeholders acknowledged the utility of this tool in addressing an acknowledged gap in group function reflection in PBL.

**Supplementary Information:**

The online version contains supplementary material available at 10.1186/s12909-023-04726-y.

## Background

### PBL and group function

Problem-based learning (PBL) in health professional education is a distinctly collaborative, socially dynamic pedagogy that is undertaken in small student groups [1], and the ability of the group to function synergistically is vitally important [2]. PBL groups tackle loosely-structured case problems [3], engaging in complex problem-solving events such as discussing the facts and information gaps of the case, forming hypotheses about the problem, identifying the group’s own learning objectives and issues, and critically synthesizing acquired knowledge [3]. In a heavily self-directed process, PBL groups are responsible for actively co-constructing their own PBL experience [4].

When leveraged appropriately, the interpersonal component of PBL presents a powerful mechanism to enrich learner experiences and support higher-order learning outcomes [2, 5], such as the development of life-long learning and clinical reasoning skills [6]. Students working in groups have an exquisite opportunity to tap into the diverse perspectives and learning processes of their peers [7, 8], to challenge their articulation of complex competencies through comparison and conflict [4], to enhance their appreciation for the learning material through engaged discussion [9, 10], and to disperse the cognitive load associated with unstructured learning amongst group members in order to grapple with problems too challenging for any individual learner [9]. Groups have thus been described as learning tools for facilitating complex cognition in professional training [4]. By contrast, an inability to capitalize on this cooperative learning leaves the potential of the group unrealized, degrading the group back into its parts— a collection of students learning together, individually [5, 11, 12].

In line with general social psychology research, it is sufficient to summarize that groups working well together can typically achieve more than individuals, but groups do not always function efficiently [12]. In practice, PBL groups differ in quality at baseline. Some groups are able to come together very naturally from the get-go and engage in collaborative work, while others are not. These differences in group function may be dependent on the gelling of a combination of individual student traits, tutor facilitation styles or abilities, and curricular or other contextual factors [2].

### Reflective learning in PBL groups

Regardless of baseline function, groups, like individuals, possess a capacity for improvement and growth. Such transformation may be achieved through an ongoing critical analysis, regulation, and development of the group’s collaborative learning processes and conditions [4, 13]. This reflective practice for which the group is responsible has been described as a “circularity between action and reflection” [14], and demands the re-examination of previous experiences to generate meaningful change in future experiences [4]. Theorists suggest that reflective practice consists of three components: reflection-before-action, reflection-in-action, and reflection-on-action [15]. These components focus on prospective expectations and intentions, real-time critical thinking, and retrospective examination of consequences, respectively, to the end of progressive cognitive and behavioral self-improvement [15]. Reflection is thus at once the key to group evolution and a core life-long learning skill important to students’ deep and meaningful growth as learners and medical practitioners [16–19].

The “double loop” learning of medical education emphasizes the role of metacognitive reflection in professional development in addition to the practical acquisition of knowledge and skills [15, 20]. Health professions PBL is thus designed with structures in place to support reflective practice for groups. Most universally, tutors are present in PBL tutorials to help scaffold group process through facilitation and feedback, without aim to contribute content expertise [9, 21]. Some institutions additionally implement formative or summative curricular components dedicated to group evaluation [22, 23], which necessitates some degree of reflection on group performance.

### Challenging and improving group reflection

Reflection around group function faces a great number of challenges. Predominantly, reflection is often neither easy nor intuitive [24]. Meaningful reflective practice is both time and resource intensive and can be uncomfortable [25], and these cognitive demands may be exacerbated when the reflection is unstructured or unpracticed. Without direction on how to focus self-reflective efforts effectively, conversations around group function become fruitlessly taxing and are thus neglected in favor of concerns around content learning, examinations, or grades [2, 11, 24–26].

To channel efficient reflection, students must first possess some degree of knowledge on how to reflect, then obtain the time and motivation needed for the reflection to occur [16, 24, 26, 27]. However, PBL students arrive into their groups with variable levels of prior knowledge and ability to engage in behavioral reflection in group settings [24]. It has been identified that many students and tutors are unaware of what components are important to effective group functioning [11], or hold differing views [28]. There is also often a lack of framework for how to contribute to group improvement [11]. Conceivably, group function improvement is limited when key players have a poor understanding as to what they are reflecting on [25, 26]. There is a perceptible need to create some guidance for thought and conversation around group function in PBL to help orient stakeholders towards effective reflection [25, 29].

Curricular performance measurement tools are known to assist the scaffolding of ongoing reflection through the provision of direction and documentation [23, 26], helping students to initiate inquiry, maintain engagement, and address misconceptions in their learning processes [7]. Such a tool for PBL group function can assist with the creation of both language and structure around this topic, and may be important to closing the “double loop” of reflective learning [15], thereby improving PBL attitudes and professionalization outcomes. The role of using tools to organize attention around core constructs for stakeholders has been done in other contexts, such as debriefing simulations [30]. An analogous tool for group function reflection in PBL, to help stakeholders start thinking about group function in categories and provide some behavioral definitions for relevant constructs, may be valuable. While a variety of scales for measuring group function exist [2, 22], these have been framed for assessment rather than reflection, and there is not a single tool that has widespread adoption across institutions [22]. Accessible reflection-oriented tools for this context are currently missing from the literature.

### Study purpose

The purpose of this study was to develop a structured measurement tool for group function in PBL, with aims to help students and tutors anchor their efforts towards group function reflection and improvement around core constructs.

## Methods

**Fig. 1:**
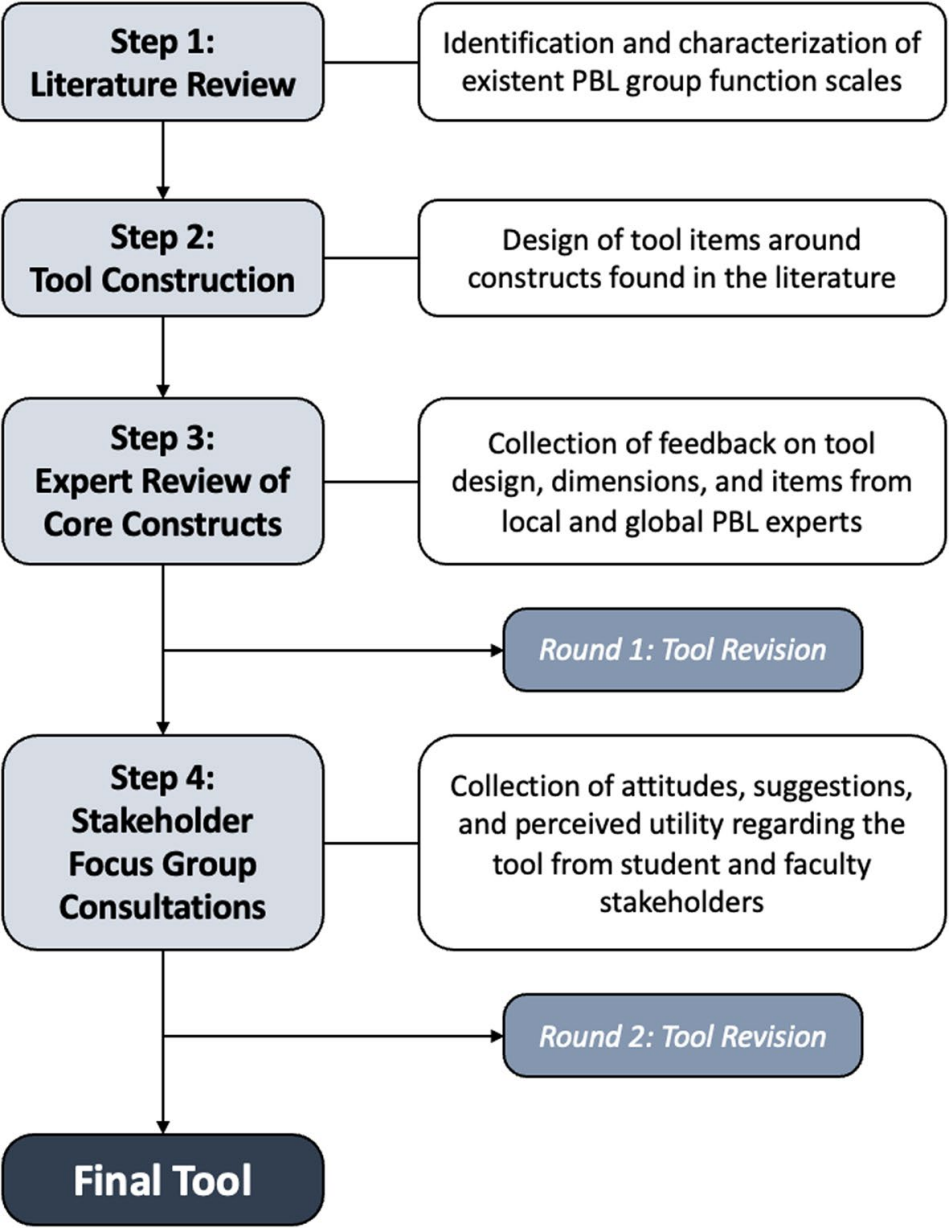
Flow diagram of study design

This study was primarily based from McMaster University and adopted a four-step, mixed-methods developmental process (Fig. 1)*.* Step 1 involved a literature review of existent scales for measuring group function in PBL. Core constructs identified from Step 1 were extracted and organized to design a new comprehensive tool in Step 2. The tool was vetted for face validity by a panel of PBL experts in Step 3, and further assessed for engagement and utility in several rounds of stakeholder focus groups in Step 4. The tool underwent two rounds of revisions, first following the expert panel review, then following focus group discussions.

### Step 1: Literature review

Published literature was consulted to identify and aggregate existent scales assessing components related to group function. Scales were included if they measured aspects related to group function or to individual student contributions towards group function. Scales intended to evaluate the PBL curriculum or tutor performance were also included if their constructs were organized around the contribution of the curriculum or tutor to specific aspects of group function. Scales for curricular or tutor evaluations without a focus on group function were excluded. Additionally, scales for which the full original or modified tool cannot be found were excluded to avoid the inappropriate overrepresentation of sample constructs.

Scales encountered during a previous scoping review [2] that met the above inclusion criteria were flagged, investigated for the parent development article where applicable, and included in this study. A further keyword search in two databases, Medline and CINAHL, was performed in May 2022 to identify any additional relevant scales.

All included scales were extracted for the following information: author, year, name of scale, intended assessor group (self-report, group, tutor eval student), evaluation object (group, individual student, tutor), design purpose (research study, curricular implementation), number of items, items/questions (listed), thematic factors (as identified by parent article; listed), theoretical framework for “good group outcome”, and additional scale characteristics.

### Step 2: Tool construction

Overlapping constructs extracted from the scales identified in the literature informed the creation of items for this new tool. The tool borrowed its format of organizing items around core constructs from a Debriefing Assessment for Simulation in Healthcare (DASH) tool [30]. The new tool’s dimensions were constructed through reflexive comparison of categories in an iterative process [31]. All decisions regarding the format, organization, rating scale, and overall presentation of the tool were decided by discussion and consensus between the first author and PI.

### Step 3: Expert review of core constructs

The first rendition of the tool was emailed to a panel of local and global PBL experts in October 2022 for initial engagement and feedback. These experts were identified by the PI and defined as individuals who have substantial lived experiences tutoring or designing PBL curricula, or those who have published extensively on PBL. The experts were asked to comment on the face validity and language of the original tool, and to make any additional suggestions. Comments were compiled and used for first-round editing of the tool.

### Step 4: Stakeholder focus group consultations

Stakeholders were defined as those who have lived experience actively contributing to group function in PBL. These included McMaster affiliated and externally affiliated faculty members who engage in PBL as tutors, educators, or researchers, as well as medical students undertaking McMaster’s pre-clinical PBL curriculum. Faculty and student stakeholders were separately invited to provide their comments on the tool’s constructs, language, and perceived utility in focus groups. The input of stakeholders was especially important since they belonged to the intended user populations for the tool and could provide valuable insights informed by their lived experiences working in, or with, PBL groups.

Recruitment and conducting of focus groups took place between November 2022 and February 2023. Participants were identified by snowball sampling and recruited by email invitation. Recruitment materials were specifically worded to encourage sample diversity during snowball sampling (e.g. varying years of study, favorable or unfavorable group experiences, facilitation by different tutors). Faculty and student participants were offered honorariums for their time and contribution, valuing $50 or $25, respectively.

Focus groups were held virtually over Zoom, a digital conferencing platform, at times of mutual convenience. Consent forms were emailed to participants prior to their scheduled focus group, allowing them time to ask questions and to provide signatures. A demographics survey was also emailed to all participants to characterize the sample. Focus groups were semi-structured, where discussions were stimulated by the presentation of the tool and guiding questions (Additional file 1: Appendix A). Focus groups lasted 60-min or until the discussion was saturated and no new ideas emerged. Faculty groups were conducted by the first author and PI. Student groups were conducted by the first author alone to minimize the influence of any power dynamics caused by the PI’s faculty appointment in McMaster’s medical school [32].

Focus groups were audio recorded and transcribed verbatim. Anonymized transcripts were coded and analyzed by three researchers (AL, MM, MS), following a directed content analysis methodology [33]. Transcripts were first read for familiarization, then re-read to deepen understanding. Initial codes pertaining to group function constructs were deductively developed based on domains identified in the literature review. Additional codes around practical tool recommendations were inductively identified. The research team met after each one to two focus groups to discuss participant comments and thematic findings. These were used to inform a second round of editing of the tool.

### Reflexivity

Subjectivity is integral to co-construction of data and meaning in qualitative research [32]. Ongoing critical examination of the influence of researcher identities and methodologies is important for maintaining rigor through the research process [32]. This author team is comprised of a diverse group of researchers with a wide range of academic, professional, and personal experiences. All have an interest in medical education research, with a focus on lifelong learning processes and pedagogical efficacy. AL (first author) is a fourth-year undergraduate Health Sciences student with experience as both a student and peer-tutor in group-based, self-directed learning. She has also published previous research around PBL group function. MM is a first-year medical student, with lived experience working in PBL groups under a medical curriculum and an interest in this line of research. Both are new to conducting qualitative research, and thus offered critical questioning of the research methodology from naive perspectives. AK is a project officer for a group of health professions education researchers and a former elementary school teacher. She is familiar with a variety of research processes and workflows and was involved as a resource manager and process consultant. MS (PI) is an experienced clinician scientist. He has been involved in PBL extensively as a formally trained tutor, researcher, and educator, and has additionally taken part in the training of other PBL tutors. He offered expertise to the research process and content, from the perspective of a mentor and practicing professional in the field. The research team communicated ideas frequently through emails, shared documents, and memos on digital platforms, creating an audit trail of contributions that was revisited and discussed in several meetings.

## Results

### Step 1: Literature review

A total of nineteen scales relating to group function assessment were extracted [34–51]. Eighteen were identified from the scoping review and one from the additional database search (Additional file 2: Appendix B).

Included scales dated between 1994 and 2016. All employed Likert-type scales, ranging from three-point to eleven-point metrics. Most were purely quantitative, with only three providing a free-text field for general feedback. Most were intended for student self-evaluation (*n* = 11), while others asked for student evaluation of their peers or tutors (*n* = 6) or tutor evaluation of students (*n* = 3). Where students were the object of evaluation, some scales asked raters to evaluate the group holistically (*n* = 9) and some asked for evaluations of individuals (*n* = 7). Approximately half the scales were initially conceptualized for curricular implementation (*n* = 12), and half for exploratory research purposes (*n* = 11).

Eighteen overlapping thematic factors or constructs were represented. Through an iterative categorization process, these constructs were mapped onto four resultant dimensions: 1) Learning Climate, 2) Facilitation and Process, 3) Engagement and Interactivity, and 4) Evaluation and Group Improvement. Across the included scales, the most frequently represented constructs include ‘active participation and quality of contribution’ (*n* = 14) and ‘motivation, morale, and sense of security’ (*n* = 12). Individual scale properties and constructs are organized and displayed in Additional file 3: Appendix C.

### Step 2: Tool construction

Constructs identified from Step 1 were used to build descriptive list items, falling under the above four dimensions, to populate the new tool. The tool uses a 7-point Likert scale (1—Extremely Ineffective/ Detrimental to 7— Extremely Effective/ Outstanding) to provide an overall score for each of its four dimensions. The score is not intended to be a summation of the individual rating or check-listing of items under each dimension, but rather an overarching impression of the group’s performance for that dimension. Users are asked to provide elaborative examples, using free-text, to justify their ratings around each dimension. Additionally, users are instructed to assess the group as a whole, rather than assessing the members independently or averaging individual scores of the group. This decision honors the fact that it is the group, and not the individual, that is the smallest functional unit in a PBL classroom.

### Step 3: Expert review of core constructs

Seven PBL experts were invited to provide feedback on the preliminary tool, and five responded. Two were PBL educators with experience designing PBL curricula, two were PBL tutors with substantial experience guiding tutorials, and one was a global PBL research expert with over 40 publications around group function in PBL.

**Fig. 2:**
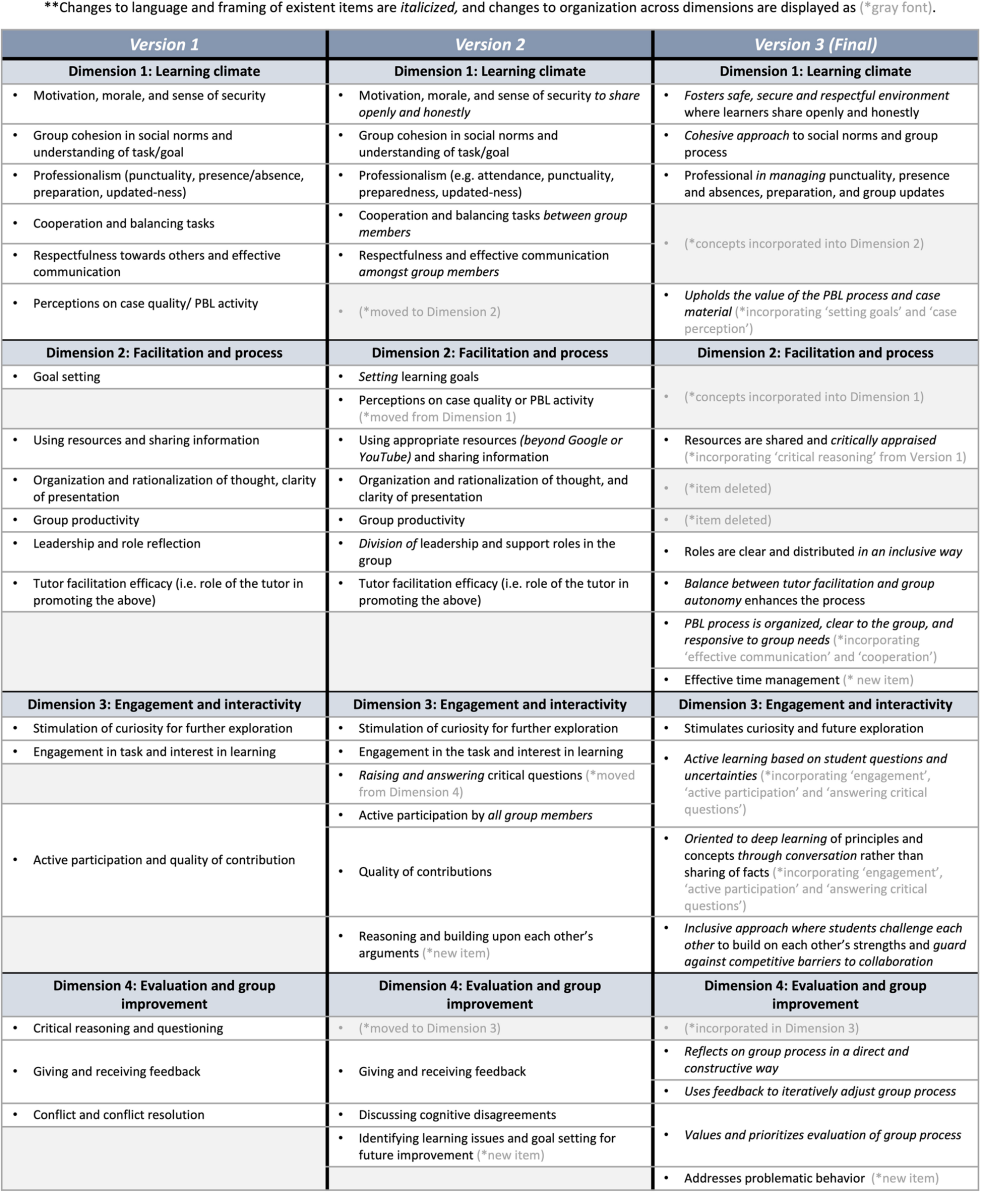
Tool revisions

All respondents indicated a keen interest for the creation of this tool. Comments were provided on the tool’s items and constructs, group function measurement practices at other institutions, and additional literature suggestions. General comments and comments specific to each dimension were compiled and used to adjust the tool. This resulted in the reorganization of some items, the addition of two items presenting additional constructs (identifying learning issues and building upon each other’s arguments), as well as descriptive elaboration of existing items. All versions of changes to the tool’s items are displayed in Fig. 2, and further informative details and illustrative sample quotes are included in Additional file 4: Appendix D.

Further discussion between the first author and PI led to the restructuring of the visual presentation of the tool. An open comments/rationale section was added to each dimension, giving space for students and tutors to elaborate on dimension-specific behaviors in relation to listed items. This qualitative component may help stimulate discussions around group function improvement in targeted areas.

### Step 4: Stakeholder focus group consultations

Two rounds each of faculty (*n* = 4) and student (*n* = 7) focus groups were conducted. All participants were affiliated with the Michael G. DeGroote School of Medicine at McMaster University, save one researcher participant, who was affiliated with the Faculty of Health, Medicine, and Life Sciences at Maastricht University. Faculty participants have had at least 5 years of experience tutoring PBL, and reported having received structured training around PBL, including faculty development courses and Master’s courses. Three of four faculty group participants have been involved in the training of other faculty members, two have published literature on PBL, and one has had experience designing PBL curricula. Student participants were first-year MD students. At the time of the focus group, students had undergone two to three PBL units under McMaster’s preclinical curriculum. Further demographics information is appended (Additional file 5: Appendix E).

Focus group participants were encouraged to share their thoughts on the development of the tool, as well as provide suggestions on its organization and language. Participants were unanimously favorable towards the tool’s creation and utility. Comments emerged around several missing constructs and unclear or vague phrasing in the tool. As a result, overall phrasing was adjusted to emphasize the active responsibility of the whole group to contribute to each item, and several key concepts were reframed and reorganized (adopting responsive group processes, creating psychological safety, active and deep learning through collaboration, challenging the self and others, and balancing tutor facilitation with group autonomy). Two items were added to the tool (addressing problematic behavior and effective use of time) and two items were ultimately deleted (group productivity and rationalization and clarity of thought). Facets of the deleted items, as they pertain to effective group function, were integrated into the descriptive elaboration of other items (Fig. 2 and Additional file 4: Appendix D).

### Final tool

**Fig. 3:**
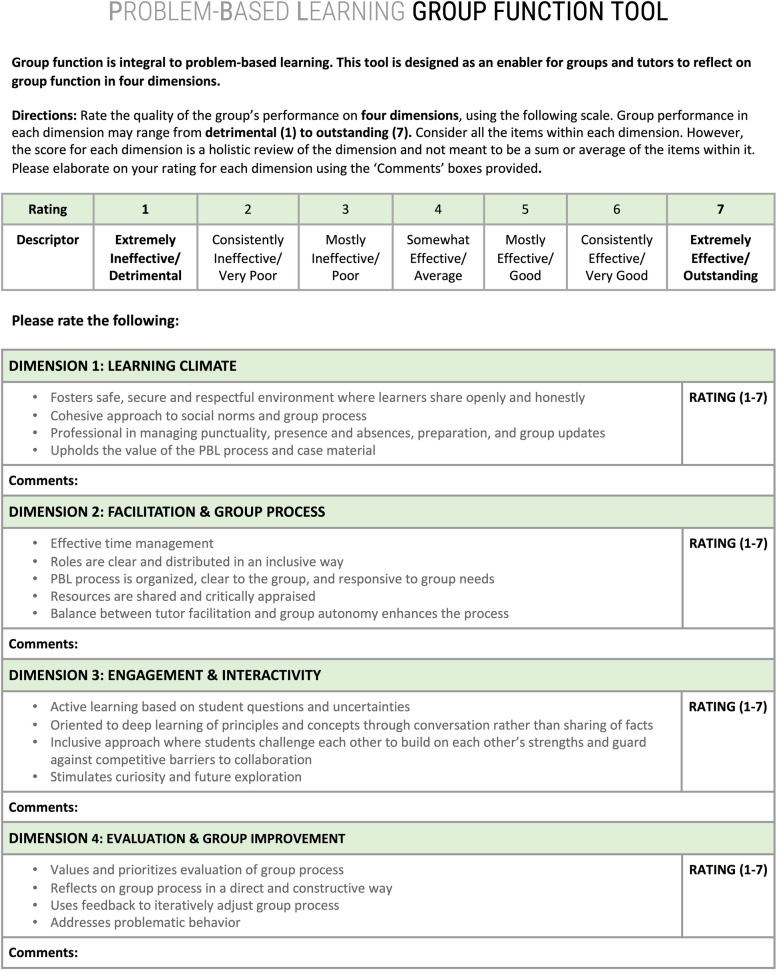
Final version of the problem-based learning group function tool

The final tool contains 17 anchoring items under the four dimensions (Learning Climate, Facilitation and Process, Engagement and Interactivity, and Evaluation and Group Improvement). Users are asked to rate each dimension holistically on a 7-point Likert scale and provide open comments. The final tool can be found in Fig. 3.

### Thematic analysis of focus groups: Tool utility

Aggregated codes from focus group discussions generated three themes and several practical concerns regarding tool utility.

Predominantly, the tool was perceived to be useful for: 1) creating space and structure for feedback processes, 2) acting as a reference or resource, and 3) serving as a written record for longitudinal benchmarking. These perceptions were echoed by both faculty and student participants. It was suggested that these functions of the tool may be particularly helpful for guiding inexperienced and poor-functioning groups.

1. *Creating space, structure, and language for feedback processes—*current procedures require McMaster medical students to provide informal feedback to their groups at the end of each PBL tutorial. Students highlighted a perceived cognitive burden and uncertainty around giving feedback, especially in relation to the lack of structure in their current feedback processes. Concerns arose around not knowing what was important to give feedback on, contributing only superficial comments, or neglecting feedback provision all together. This tool had a perceived potential for counteracting the current lack of content and implementation structure for feedback processes:
  *“In my experience, feedback is pretty informal. And sometimes it's talking about group function, group process. Sometimes it's directed at specific people and giving specific individuals feedback on their performance. Sometimes feedback doesn't even happen. […] Telling people how to do feedback in a sense with this tool would probably make feedback as a whole more effective.”* (Student 4, SG2)
  Breaking down group function into smaller, digestible dimensions and indicating behavioral concepts that may contribute to good functioning were perceived as being particularly helpful.
  It was also identified that providing honest real-time feedback is particularly difficult for groups that have not already established a sense of safety and openness towards self-reflective processes. The establishment of such an environment, in and of itself, demands a higher level of baseline group functionality that may not always be present:
  *“If the group function is not working, I don't feel comfortable to tell you that the group function is not working, which means you can't fix the function in the first place.”* (Student 1, SG1)
  When the safe environment is absent, it was suggested that a structured tool may be helpful in prescribing the time and space for facilitating feedback processes.

2) *Acting as a reference, resource, or memory aid—* Participants commented on the challenges of giving group feedback in real-time, due to memory lapses in behavioral examples or psychological phenomena such as the recency effect. An accessible visual reference was perceived by both tutors and students as helpful for keeping the important group function constructs sentient when participating in tutorials and during active group feedback processes:
  *“Sometimes during tutorial, I pick up on something that I wanna give feedback about, but I just forget about it later […] But having a framework like this allows me to remember, oh, okay, my concern was in this particular domain or in that domain of the group function process.”* (Student 2, SG1)
  Additionally, participants outlined that giving feedback is especially difficult for those who are engaging in PBL for the first time, and who may not know what constitutes good or poor group function. This tool is seen as a helpful “jumping point” for giving words to important behaviors that groups may or may not already be aware of or taking part in:
  *“I don't have experience giving feedback, so it feels like a little disingenuous and very foreign to come up with things that I could contribute in a feedback process. But if there was a reference, especially in the beginning, I could build off of that.”* (Student 5, SG2)

3) *Serving as a written record for longitudinal benchmarking*— Finally, the tool was seen as a valuable artifact to substantiate and give permanence to feedback. Having a structured document on which feedback may be provided was thought to allow retrospective assessment of group improvement in specific areas. Students can refer to their previous assessments and receive tangible acknowledgement of the growth of the group in any particular dimension over time:
  *“It's very difficult to monitor progress from one tutorial to the next on how a group function improved or declined when there's no quantification, when there's no scale. This allows us to have like a baseline, see how we perceive our group is functioning and quantitatively compare that to the future and see like, did we actually improve on these on these fronts that we express concern about?”* (Student 2, SG1)

Further quotes supporting these 3 themes can be found in Additional file 6: Appendix F.

### Implementation considerations

Additionally, student participants indicated their perspectives on several practical implementation concerns. These revolved around the reflective limitations of self-assessment, the social hesitation to give negative feedback, the desire for anonymity, and concerns for the time and cognitive demands of giving feedback:

*“The feedback, in a lot of times, it's just positive, meaning the students just verbally go around and say, ‘Today was a great session. I really enjoyed the discussion.’ [...] I sometimes worry that students might be withholding genuine, constructive feedback and thoughts because they're worried about repercussions that they're going to face from their group or from their tutor in terms of, I'm not saying like literal repercussions, like a deduction in your mark, but just the looks you're going to get. And that in itself is enough for someone to hold back from saying what they wanna say.*” (Student 2, SG1)

*“Sometimes I think that we could benefit from a bit more feedback, but I also think it's time-consuming and it's logistically challenging to do more feedback.”* (Student 4, SG2)

Again, inexperienced groups and poor-functioning groups where psychological safety is not present were particularly highlighted for consideration. Further quotes illustrating implementation concerns and considerations are presented in Additional file 7: Appendix G.

## Discussion

Group function depends upon a multitude of complex interpersonal processes, phenomena, and conditions [2]. Encouraging students’ abilities to autonomously regulate and optimize collaborative learning processes is a key component of PBL. However, poor knowledge of how to characterize ‘good’ group function hinders students’ ability to effectively reflect on and improve their own group processes [11, 25]. Tutors, when suffering from the same lack of understanding, may struggle to facilitate group reflection [11, 52]. Structural and curricular supports around group function reflection currently lacks any mechanisms to help guide thought towards important constructs in this topic. This study thus aimed to produce a tool to aid facilitation of reflective learning for PBL groups. Constructs important to group function in PBL were collated from the literature and a 17-item tool was developed to measure and practically anchor reflection in PBL tutorials. This tool organizes its core constructs in four dimensions, adding some structural scaffolding to improve reflection around group function.

This new tool is a valuable addition to PBL literature and curricula in several ways. Methodologically, the multi-phased developmental process of this tool was more rigorous than that reported for prior scales on this topic. The design of this tool was drawn from a compilation of constructs from previous tools, then triangulated by input from experts and stakeholders, giving it a comprehensive scope of what is important to group function [53]. Several prior scales presented overlapping constructs but none comprehensively addressed all aspects of group functioning. Researchers, educators, tutors, and students were all in favor of the creation of this tool, acknowledging its potential to bridge a gap in measurement methods for group function in PBL. This support from stakeholders provided evidence of the tool’s utility and relevance to current curricular practices. Areas of revision and addition to the tool based on expert and stakeholder feedback could provide insight into topics for further research, since the feedback identified gaps in previous literature regarding group function measurement. Items and ideas that were redistributed across dimensions due to debate over categorical membership may point to concepts that are most central to group function, since these mark points of intersectionality between dimensions.

Additionally, this tool was the first group function measurement tool designed to stimulate qualitative reflection in addition to quantitative assessment of performance. Many prior tools were initially developed for research contexts rather than curricular implementation, with aims to advance the literature or help curricular development rather than directly guide student reflection. Most were strictly quantitative in nature, without room to share elaborative examples. In contrast, this tool provides structure for reflection by categorizing its items without asking its users to numerically rate any individual item. Students may use the items as jumping points to generate discussion with group members and tutors, or as memory triggers to stay cognizant of group reflection throughout the PBL process. The free-text feedback fields in each dimension of the tool allow students and tutors to elaborate on their experiences and record practical examples in specific domains.

However, there are some practical concerns to keep in mind. First, though the importance of feedback and improving group function was acknowledged, both students and tutors expressed that they did not want to allocate too much of their limited tutorial time to these process discussions. How and when to administer the tool to maximize reflective efficacy in groups must be considered by PBL tutors and educators. Additionally, there may be a discussion to be had regarding the degree of responsibility that students and tutors each have towards maintaining group function, particularly when social and curricular stressors are at play. For instance, faculty participants shared that when students are uncomfortable with their group function, avoidance is generally preferred over confrontation, and tutors are left to intervene. This phenomenon is not new and has been commonly echoed in the literature [11, 52]. Student participants, on the other hand, pointed out social concerns for giving criticism, such as fear of judgement from peers. The concept of anonymity seemed to alleviate concerns around giving honest and direct feedback, and lack of anonymity was expressed as a barrier. In any case, while tutors can use the tool to set up initial expectations around group function feedback and reflection and to help keep groups on track, the bulk of responsibility for maintaining group function should fall onto the members of the group itself.

It may also be important to note that groups operate within the limits of their social and technological contexts [21], which has implications for performance measurement tools. For instance, the ability to navigate and leverage technology as sources of information (e.g. shared electronic documents, online databases) is a novel part of collaboration and professionalism training, bringing unique benefits and challenges to how groups communicate [21, 54]. Though this tool serves as a review, synthesis, and update to previous available tools, most of which are dated back by decades, the contextual dependency of group function indicates a continuous need to regularly revisit and revise tools in the future to meet advancing technological implications for group practices.

Acknowledging that time and cognitive resources are finite, process reflection will always be in competition with content learning, and formative exercises will most often likely be overshadowed by summative assessments [11, 25]. This tool may help motivate reflection in some major ways. First, in providing structure to organize thought around group function, the tool may alleviate some of the cognitive demands associated with process reflection [29] and thus reduce students’ reluctance towards engaging in reflective practices [27]. Second, having a formalized tool for group function reflection may help students take group function more seriously and devote more resources towards it [24, 26, 27]. In all, this tool shows potential in prompting students to take action and ownership of their own group function.

## Conclusion

This study developed a tool to help reconcile a gap in students’ and tutors’ understandings of what is important to group functioning in PBL and, subsequently, how to improve group function. The tool’s theoretical grounding was well supported by previous literature and PBL experts, and its practical utility was appreciated by both student and faculty stakeholders. The tool was perceived to be useful for providing structure to group feedback processes, acting as a resource to guide group feedback processes in tutorials, and facilitating longitudinal benchmarking of group progress in specific domains. This tool may provide groups with the necessary scaffolding to facilitate effective reflection on the interpersonal components of PBL.

This study has some limitations. While the multi-phase process adds rigor, it must be acknowledged that most of the contributions to this study came from an undergraduate medical program community at one institution. These perspectives may not be generalizable to other communities or contexts. Additionally, the study sample may have been overrepresented by students and tutors who embraced PBL pedagogy or enjoyed their PBL experiences, since they would be more likely to participate in PBL research than those who did not. However, during the focus groups, participants highlighted both positive and negative group experiences. Finally, focus group coding and thematic analysis processes are fundamentally dependent upon researcher interpretations. Reflexivity practice and the involvement of multiple researchers in the coding and analysis process aimed to diversify interpretations and minimize bias.

Tutors are encouraged to implement the use of this tool into their PBL tutorials to orient students to group function in PBL and subsequently guide formal reflection and feedback processes. The tool may additionally be made available to students and tutors as a digital or physical resource to prompt informal individual or group reflection. Following successful circulation and implementation, future studies may report on the user satisfaction and psychometric properties of the tool. The impact of tool usage on learners’ behavioral, academic, and professional outcomes may also be explored for groups with varying degrees of PBL familiarity and competency.

### Supplementary Information

**Additional file 1: Appendix A.** Focus Group Guiding Questions.

**Additional file 2: Appendix B.** Literature Review Process.

**Additional file 3: Appendix C.** Characterization of PBL Group Function Scales Available in the Literature.

**Additional file 4: Appendix D.** Details Supporting Tool Revisions. 

**Additional file 5: Appendix E.** Focus Group Demographics Surveys.

**Additional file 6: Appendix F.** Focus Group Perspectives on Tool Utility.

**Additional file 7: Appendix G.** Focus Group Perspectives on Tool Implementation Considerations and Concerns. 

## Data Availability

The raw data used and/or analyzed in the current study are available from the corresponding author upon reasonable request.

## Abbreviation

PBL: Problem-based learning
