# Supplementary figures and images for: Measuring group function in problem-based learning: development of a reflection tool
Source: BMC Med Educ. 2023 Oct 10;23:745. doi: 10.1186/s12909-023-04726-y (PMC10566193; doi:10.1186/s12909-023-04726-y)

Appendix A: Focus Group Guiding Questions


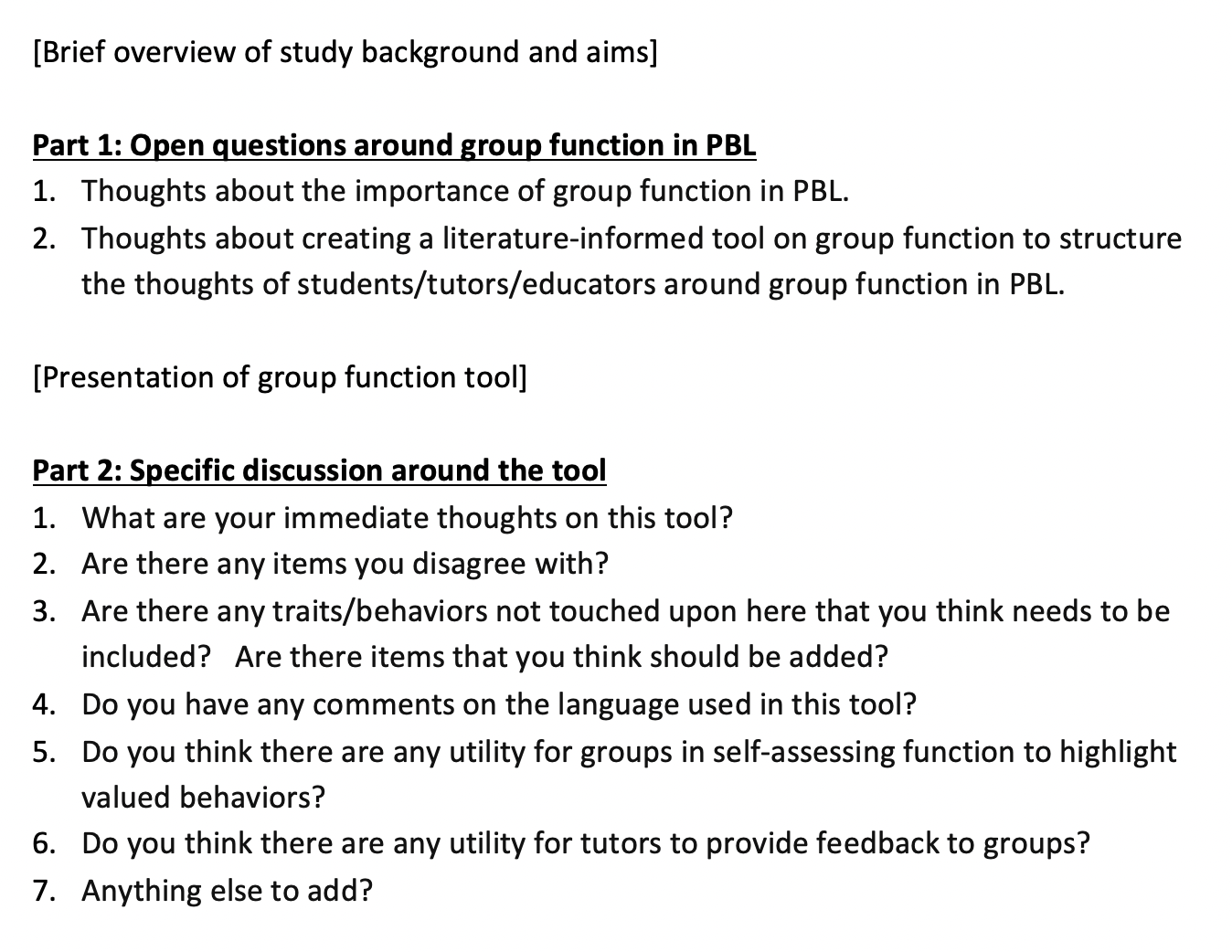

Supplement: Supplementary file 1 — Additional file 1: Appendix A. Focus Group Guiding Questions. [file 12909_2023_4726_MOESM1_ESM.docx]

Appendix B: Literature Review Process


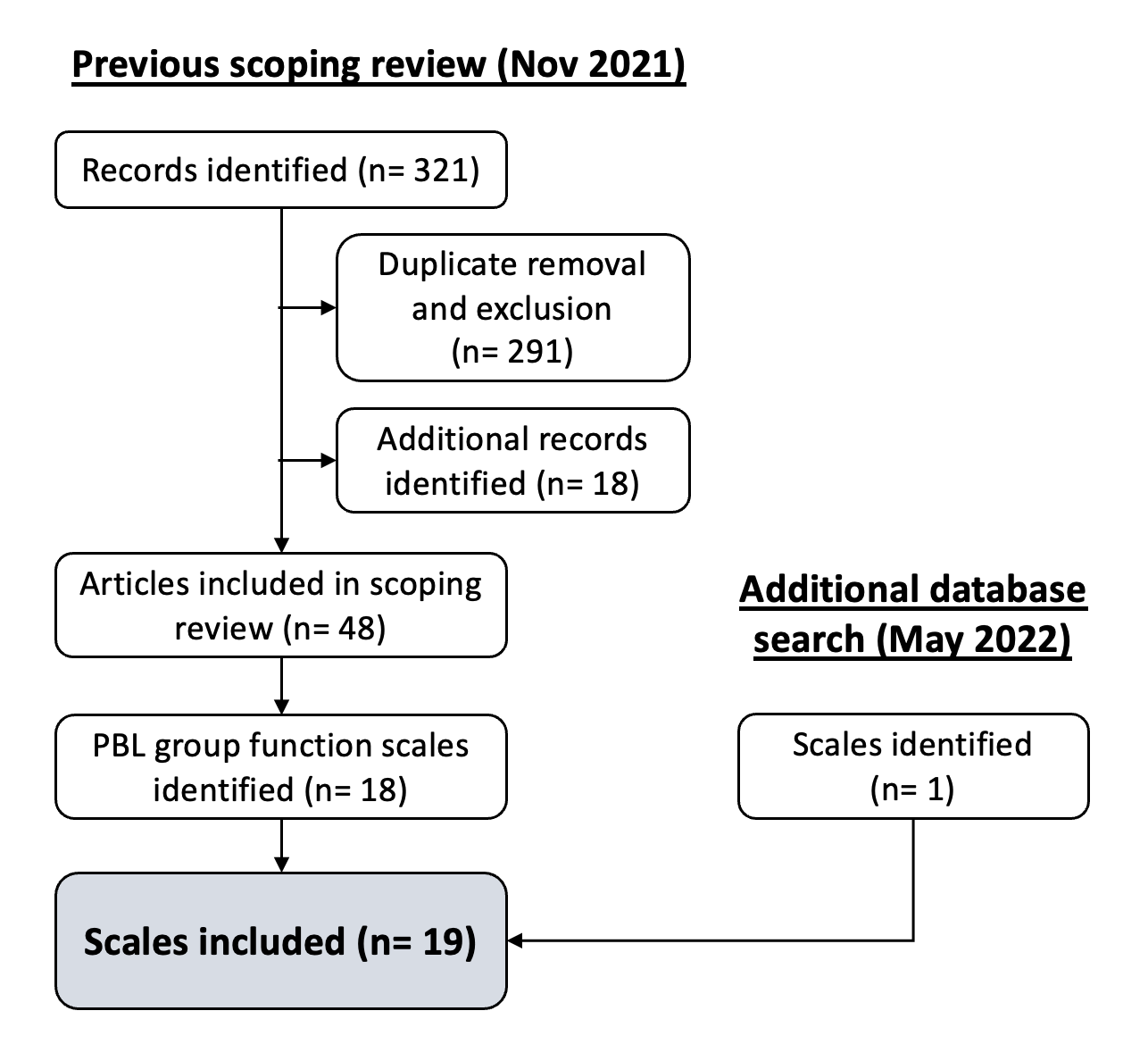

Supplement: Supplementary file 2 — Additional file 2: Appendix B. Literature Review Process. [file 12909_2023_4726_MOESM2_ESM.docx]

Appendix D: Details Supporting Tool Revisions


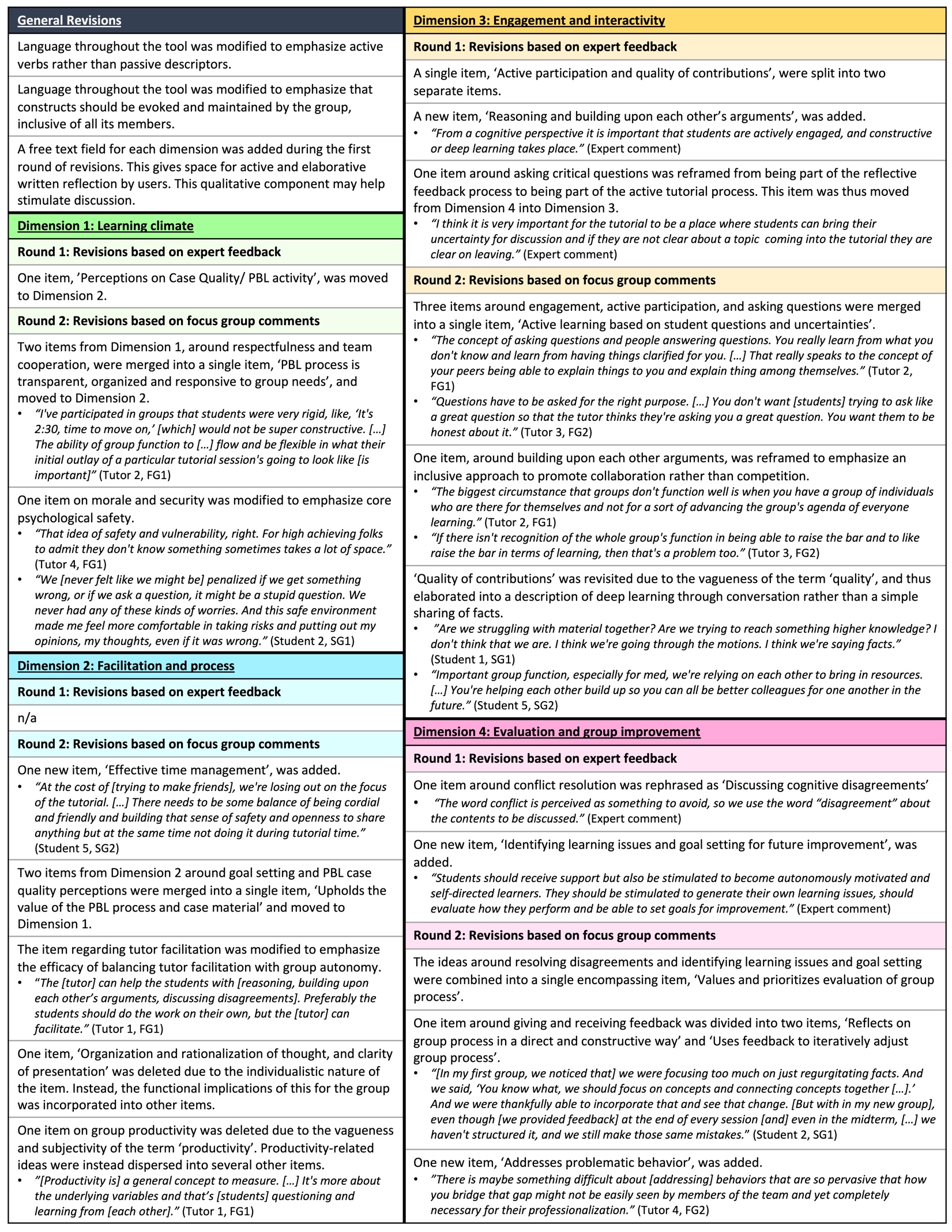

Supplement: Supplementary file 4 — Additional file 4: Appendix D. Details Supporting Tool Revisions. [file 12909_2023_4726_MOESM4_ESM.docx]

Appendix E: Focus Group Demographics Surveys


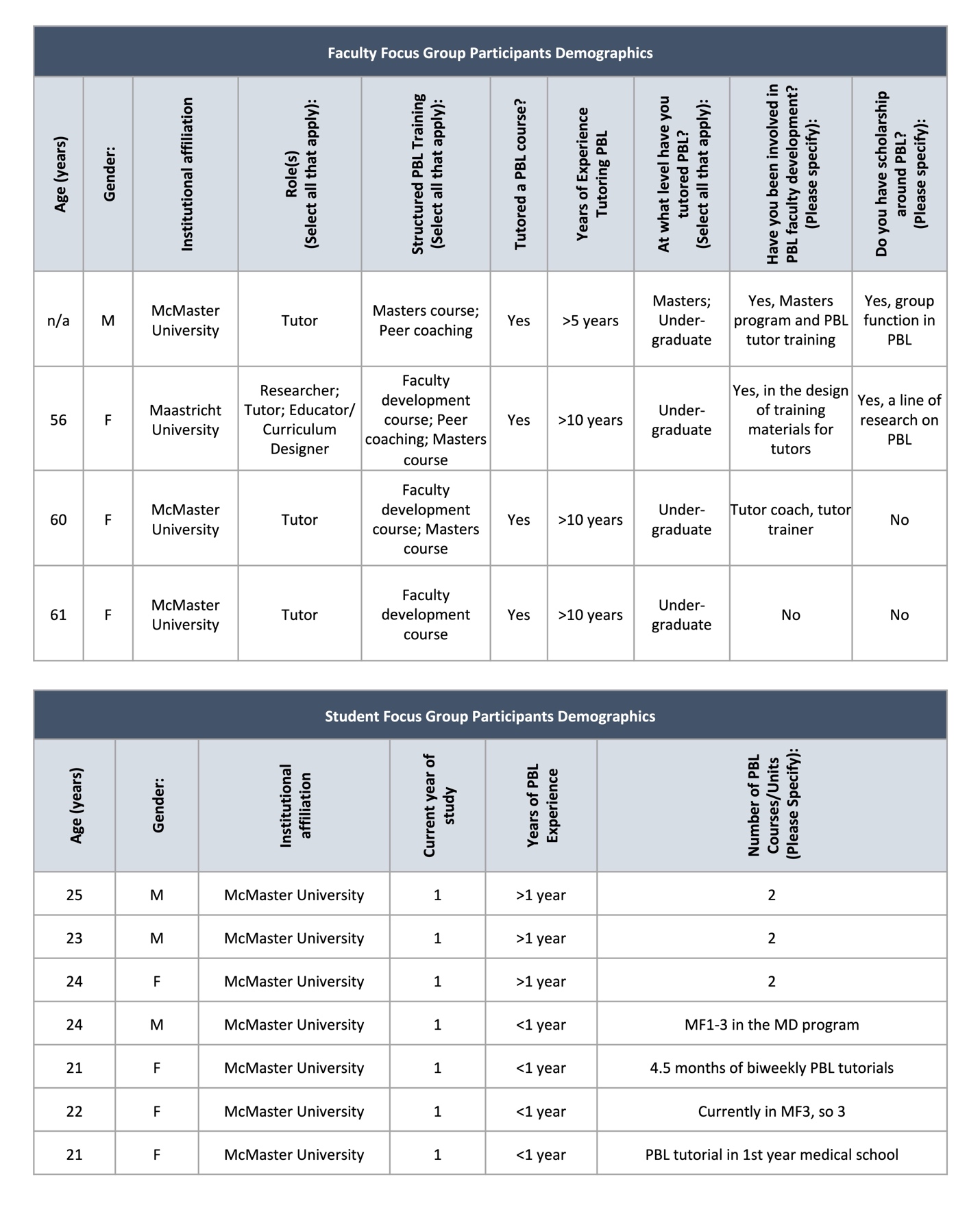

Supplement: Supplementary file 5 — Additional file 5: Appendix E. Focus Group Demographics Surveys. [file 12909_2023_4726_MOESM5_ESM.docx]

Appendix F: Focus Group Perspectives on Tool Utility


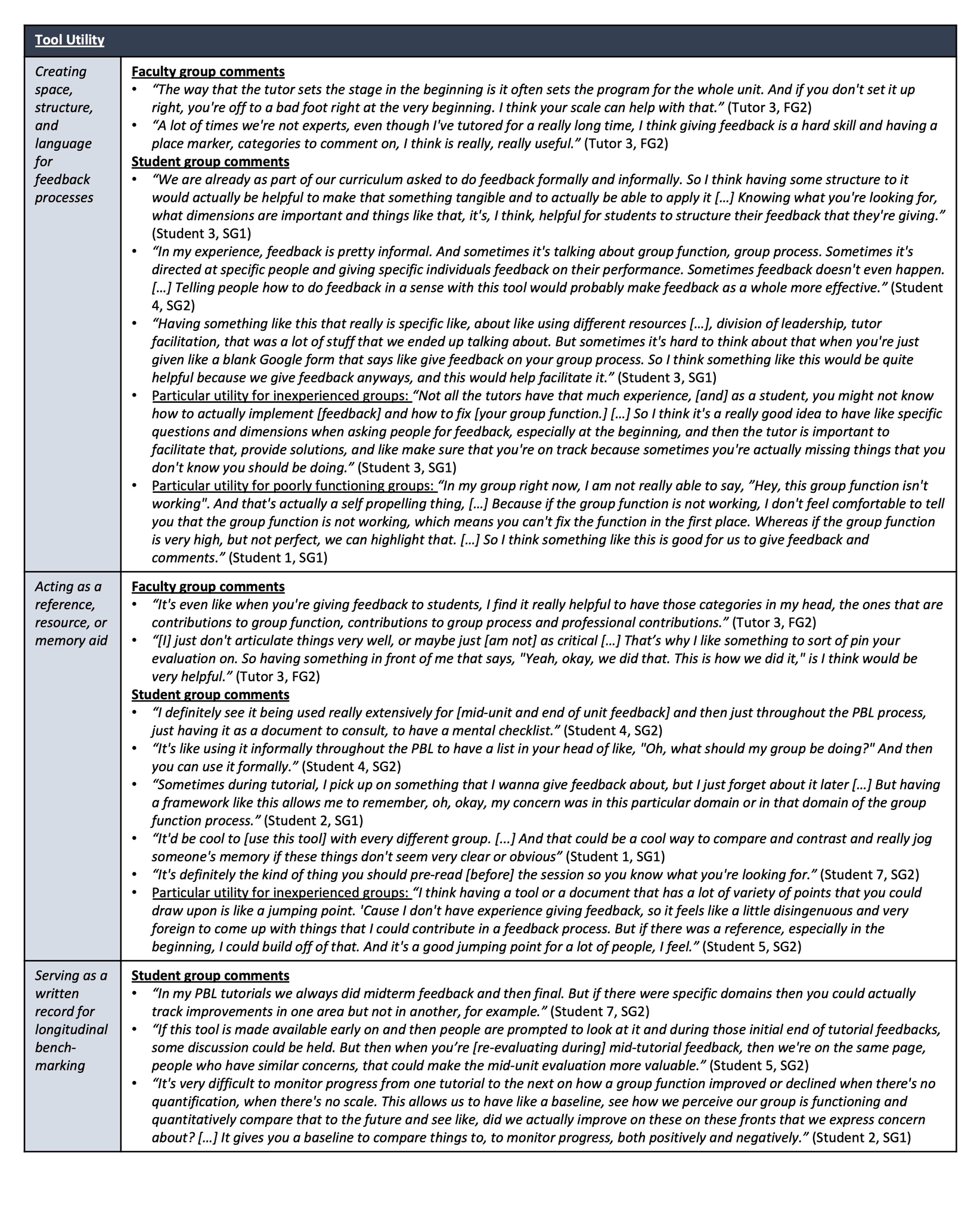

Supplement: Supplementary file 6 — Additional file 6: Appendix F. Focus Group Perspectives on Tool Utility. [file 12909_2023_4726_MOESM6_ESM.docx]

Appendix G: Focus Group Perspectives on Tool Implementation Considerations and Concerns


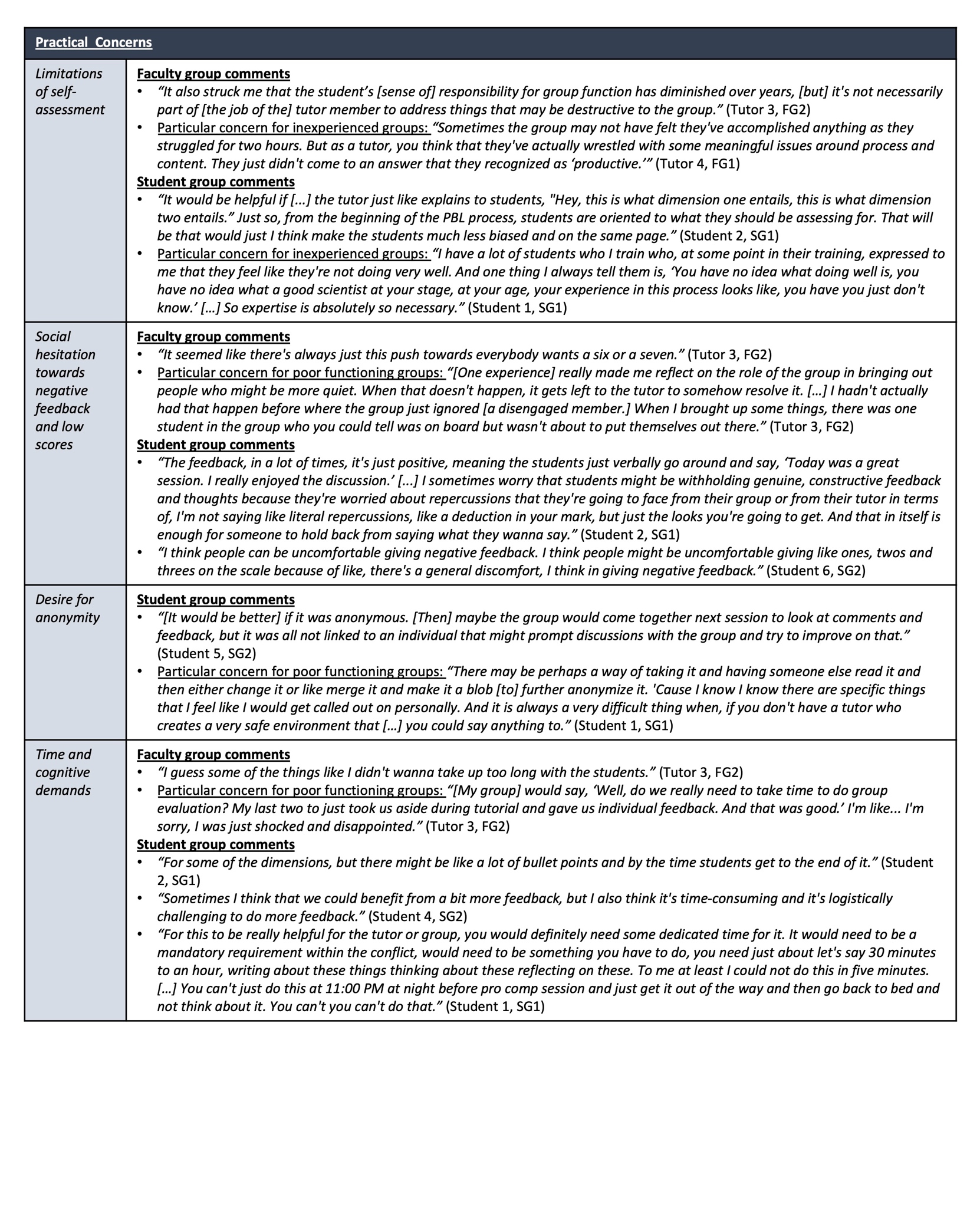

Supplement: Supplementary file 7 — Additional file 7: Appendix G. Focus Group Perspectives on Tool Implementation Considerations and Concerns. [file 12909_2023_4726_MOESM7_ESM.docx]
