# Supplementary material for: Measuring group function in problem-based learning: development of a reflection tool
Source: BMC Med Educ. 2023 Oct 10;23:745. doi: 10.1186/s12909-023-04726-y (PMC10566193; doi:10.1186/s12909-023-04726-y)
Supplement: Supplementary file 3 — Additional file 3: Appendix C. Characterization of PBL Group Function Scales Available in the Literature. [file 12909_2023_4726_MOESM3_ESM.docx]

Appendix C: Characterization of PBL Group Function Scales Available in the Literature
